# Accelerating 3D printing of pharmaceutical products using machine learning

**DOI:** 10.1016/j.ijpx.2022.100120

**Published:** 2022-06-09

**Authors:** Jun Jie Ong, Brais Muñiz Castro, Simon Gaisford, Pedro Cabalar, Abdul W. Basit, Gilberto Pérez, Alvaro Goyanes

**Affiliations:** a Department of Pharmaceutics, UCL School of Pharmacy, University College London, 29-39 Brunswick Square, London WC1N 1AX, UK; b IRLab, CITIC Research Center, Department of Computer Science, University of A Coruña, Spain; c IRLab, Department of Computer Science, University of A Coruña, Spain; d FabRx Ltd., Henwood House, Henwood, Ashford TN24 8DH, UK; e Departamento de Farmacología, Farmacia y Tecnología Farmacéutica, I+D Farma (GI-1645), Facultad de Farmacia, iMATUS and Health Research Institute of Santiago de Compostela (IDIS), Universidade de Santiago de Compostela, 15782, Spain

**Keywords:** Additive manufacturing of pharmaceuticals, Manufacture of medicinal products, Fused filament fabrication and Fused deposition modelling, 3D printed drug products and medicines, Printing medical devices and implants, Artificial intelligence and digital health, Material extrusion and drug delivery systems

## Abstract

Three-dimensional printing (3DP) has seen growing interest within the healthcare industry for its ability to fabricate personalized medicines and medical devices. However, it may be burdened by the lengthy empirical process of formulation development. Active research in pharmaceutical 3DP has led to a wealth of data that machine learning could utilize to provide predictions of formulation outcomes. A balanced dataset is critical for optimal predictive performance of machine learning (ML) models, but data available from published literature often only include positive results. In this study, in-house and literature-mined data on hot melt extrusion (HME) and fused deposition modeling (FDM) 3DP formulations were combined to give a more balanced dataset of 1594 formulations. The optimized ML models predicted the printability and filament mechanical characteristics with an accuracy of 84%, and predicted HME and FDM processing temperatures with a mean absolute error of 5.5 °C and 8.4 °C, respectively. The performance of these ML models was better than previous iterations with a smaller and a more imbalanced dataset, highlighting the importance of providing a structured and heterogeneous dataset for optimal ML performance. The optimized models were integrated in an updated web-application, *M3DISEEN*, that provides predictions on filament characteristics, printability, HME and FDM processing temperatures, and drug release profiles (https://m3diseen.com/predictionsFDM/). By simulating the workflow of preparing FDM-printed pharmaceutical products, the web-application expedites the otherwise empirical process of formulation development, facilitating higher pharmaceutical 3DP research throughput.

## Graphical abstract

**Figure:**
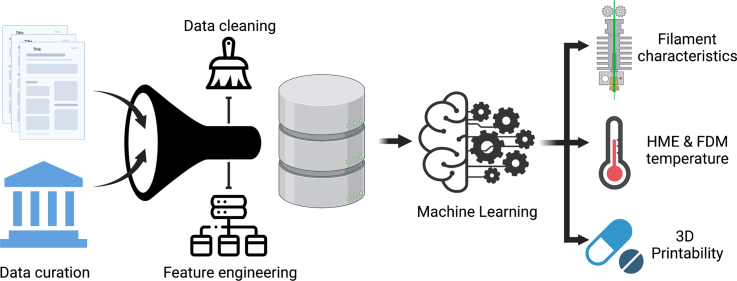

## 1 Introduction

3D printing (3DP), or additive manufacturing, is a contemporary manufacturing technique by which a 3D object is fabricated layer-by-layer based on a computer-aided design (CAD) model. According to the American Society for Testing and Materials (ASTM), 3DP technologies are sub-divided into 7 categories: material extrusion, material jetting, binder jetting, powder bed fusion, sheet lamination, vat photopolymerization, and directed energy deposition (American Society for Testing and Material, 2021). Amongst these, Fused Deposition Modelling (FDM) – a type of material extrusion 3DP – is the most common technique due to its low cost, simple operation, and non-toxic feedstock (Cailleaux et al., 2021; Elbadawi et al., 2021c). In FDM 3DP, filaments are first made by hot melt extrusion (HME), wherein a mixture of powders is poured into an extruder that applies heat and shear stress and extrudes the molten material through a nozzle to form a filament (Cailleaux et al., 2021; Fanous et al., 2021). The filament is subsequently fed into an FDM 3D printer, where it is heated again through a nozzle and deposited on a build plate, tracing a 2D pattern as pre-defined by the uploaded CAD model. The molten filament is deposited layer-by-layer, until the entire 3D geometry is built. The simplicity and versatility of FDM 3DP has led to its adoption in numerous industries, including the healthcare sector (Dumpa et al., 2021; Seoane-Viaño et al., 2021). Applications of FDM 3DP in the medical field include patient-specific organ replicas for surgery preparation, surgical instruments, custom-made prosthetics, personal protection equipment, and pharmaceuticals (Aimar et al., 2019; Fan et al., 2020; Kholgh Eshkalak et al., 2020; Martin et al., 2021).

Pharmaceutical 3DP has garnered considerable research interest for its ability to fabricate medicines with size, geometry, release profiles and dose tailored to an individual's specific clinical needs (Capel et al., 2018; Seoane-Viaño et al., 2021). Specifically, the simplicity and low cost of FDM 3DP has led to the general view that it may be the technology to be clinically adopted to produce personalized medicines. Enthusiasm towards FDM 3DP amongst pharmaceutical researchers has been demonstrated by its use to manufacture a range of drug delivery devices, including 3D printed tablets (Printlets) (Bogdahn et al., 2021; Figueiredo et al., 2022; Isreb et al., 2019; Melocchi et al., 2021; Oladeji et al., 2022; Pereira et al., 2020; Shi et al., 2021; Tranová et al., 2022; Windolf et al., 2022), gastro-retentive tablets (Zhao et al., 2022), microneedles (Wu et al., 2021), and patient-specific devices (Arany et al., 2021; Carlier et al., 2021; Eleftheriadis and Fatouros, 2021; Eleftheriadis et al., 2020; Haddow et al., 2021; Liang et al., 2018; Saviano et al., 2022). While interest in pharmaceutical 3DP continues to grow, with pharmaceutical companies such as Aprecia and Triastek investing in the technology, progress is arguably hampered by the empirical process of formulation development (Elbadawi et al., 2021b; Seoane-Viaño et al., 2021). The entire process of HME and FDM 3DP typically involves iterative adjustments to the formulation composition and/or numerous printing parameters such as the extrusion speed and temperature, the printing speed and temperature, the layer height, the percentage infill, and the platform temperature (Đuranović et al., 2021; Govender et al., 2021; Henry et al., 2021; Zhang et al., 2021). Given the multifactorial nature of HME and FDM 3DP, conventional systemic methods of evaluating each input variable on printing success (i.e., based on design of experiments) can be time-consuming. The large amount of data derived from almost a decade of pharmaceutical 3D printing research contains critical yet convoluted information that could accelerate formulation development if structured and unraveled (Crișan et al., 2022; Elbadawi et al., 2021c; Manini et al., 2022).

Machine learning (ML) is an application of artificial intelligence (AI) that enables pattern recognition from large and complex datasets. ML has garnered considerable interests and accolades in recent years owing to its success in affording actionable insights across disciplines that humans and conventional strategies struggle or fail to provide (Kourou et al., 2015; Libbrecht and Noble, 2015; Sarker, 2021). For instance, Google DeepMind's AlphaFold predicts the 3D morphology of proteins based on their amino acid sequence, providing computational biologists time and resource savings compared to conventional approaches such as X-ray crystallography (Callaway, 2020). In healthcare, ML-powered products are increasingly receiving regulatory clearance, with a 2020 study finding that AI/ML systems are winning approval from the US Food and Drug Administration (FDA) at an accelerating rate (Benjamens et al., 2020; Rajpurkar et al., 2022). The transformative effect that ML has had on other industries has prompted the pharmaceutical industry to identify opportunities to re-invent traditional time-consuming processes in bringing medicines into market (Abramov et al., 2022; Elbadawi et al., 2021a; Kolluri et al., 2022; Lou et al., 2021; Paul et al., 2021; Thomas et al., 2021; Yang et al., 2019).

In our previous studies, we reported an AI-based web application, named *M3DISEEN*, that employed five ML techniques to accelerate the development of HME and FDM formulation development. The ML models utilized a dataset comprising 614 drug-loaded formulations produced by researchers from University College London - School of Pharmacy to predict three parameters: processing temperatures (extrusion and printing temperatures), feedstock characteristic, and printability (Elbadawi et al., 2020). The dataset, while containing a sizeable amount of negative data, was limited by its small size. To obtain a larger dataset, we extracted and utilized 980 3D printed formulations from published literature to predict the aforementioned parameters and the drug release profiles of the printed devices (Muñiz Castro et al., 2021). Simulations of drug release profiles were found to be accurate and is expected to provide significant time saving as pharmaceutical researchers could now optimize their product design without having to physically print and test them – a process that would have taken days for each iteration. While learning performance improved from our initial work, the models were hampered by positively biased reporting in the literature data, unsurprisingly given the motivation of researchers to only publish good results. The lack of negative data is not ideal for training ML models, as they tend to learn only from a single class. Therefore, combining the literature-mined dataset with in-house data could provide the breadth and balance necessary for optimal ML performance.

The present study aims to enhance the performance of various ML techniques, using data from in-house printing experiments and data mined from published literature, in predicting FDM 3DP processing temperatures, printability, and filament mechanical characteristics. Subsequently, an updated AI-based web application was developed utilizing the new combined dataset to provide predictions on filament mechanical characteristics, extrusion temperature, printability, printing temperature, and drug release profiles (https://m3diseen.com/predictionsFDM/).

## 2 Materials & methods

### 2.1 Data

The dataset used for the study was derived from two different sources of data. Foremost, data on in-house formulations, comprising 64 materials and 614 formulations, were used as described in the previous study (Elbadawi et al., 2020). This was supplemented with 254 materials and 980 formulations that were mined from 114 published articles between Jan 1, 2013, and November 30, 2020 (Muñiz Castro et al., 2021). The variables included within the dataset can be divided into three groups: *material variables* (described in Section 2.2), which refers to the materials used for a formulation; *process-related parameters* (described in Section 2.3), which are related either to the hot melt extrusion process or to the FDM 3D printing process; and finally, the *predicted target variables* (described in Section 2.4), which are the variables that the ML models are built to predict.

### 2.2 Feature set selection and creation

**Fig. 1:**
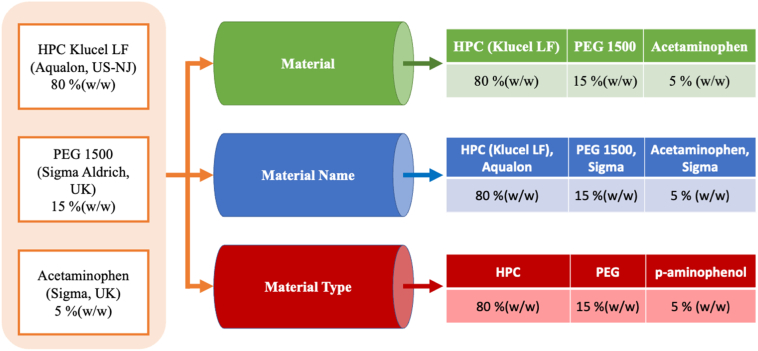
Schematic illustrating how materials were classified in the feature sets material, material name and material type.

**Fig. 2:**
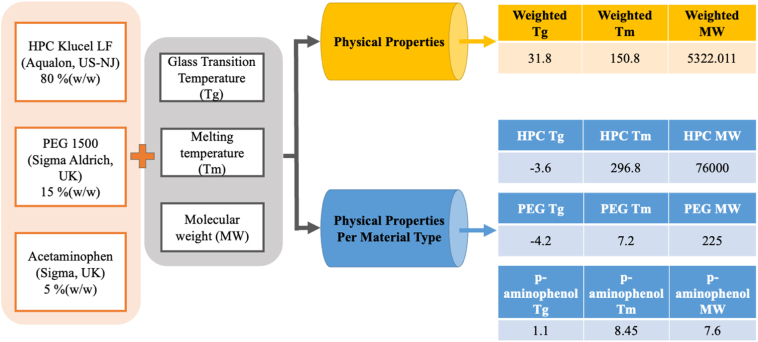
Schematic illustrating how the feature sets physical properties and physical properties per material type were created.

Five feature sets used herein were *material with company name, material, material type, physical properties,* and *physical properties per material type*. The feature sets differ in how the information about the materials used in the formulation is represented and were created as previously reported (Elbadawi et al., 2020). *Material with company name* uses the weight fraction of each material as input (Fig. 1). On the other hand, *Material* treats same materials from different suppliers as the same material; in other words, materials are grouped by their name, disregarding the supplier company. The feature set *material type* also groups materials in the same way but by their chemical structure. The *physical properties feature set* uses the weighted glass transition temperature, melting temperature and molecular weight as inputs (Fig. 2). The values for the properties are computed as a weighted average using the weight fraction and the properties of each material used in each formulation. In cases where the physical property of the material is unknown, the weighted average is calculated using only the weight fraction and the properties of the remaining materials that make up the formulation. The final feature set is a combination of *physical properties* and *material type*, where the materials are grouped by their chemical structures and the input is the weighted physical properties for each group. Schematics illustrating the creation of the feature sets are presented in Fig. 1, Fig. 2.

### 2.3 Process-related parameters

Information on the extrusion and the printing process were also reflected in the dataset. This includes printing parameters such as the extrusion speed or the printing speed as well information about the equipment such as the type of extruder used, or the brand of the printer used. These variables are described in Table 1. In addition to being process-related parameters, both *extrusion temperature* and *printing temperature* are also predicted target variables.

**Table 1:** Summary of the process-related variables.

Variable	Description
Extruder brand	Model and company of the hot melt extruder used.
Extruder type	Number of screws that are inside the hot melt extruder chamber (i.e., single or twin-screw extruder).
Extrusion Temperature (°C)	Temperature at which hot melt extrusion was conducted, as measured by the thermocouple located at the nozzle of the extruder.
Extrusion Speed (RPM)	Speed of rotation of the screws in the chamber of the extruder.
Extrusion Torque (N.cm)	The force exerted by the rotation of the extruder screws on the powder mix.
Printer Brand	Model and company of the 3D printer used.
Printing Temperature (°C)	Temperature at which the filaments are heated and extruded through the 3D printer nozzle, as measured by the thermocouple located at the nozzle of the 3D printer.
Printing Speed (mm/s)	Speed at which the printer head moves.
Platform Temperature (°C)	Temperature of the build plate on which heated filaments are deposited on and the 3D object is made.
Nozzle Diameter (mm)	Size of the orifice through which heated filaments were extruded from on the 3D printer.
Object	Type of device that was being printed (e.g., tablet, film, etc.).
Shape	General description of the 3D geometry that was printed.

### 2.4 Predicted target variables

The key parameters that the study aimed to predict were the extrusion temperature, filament mechanical characteristics, printing temperature and printability (Table 2). These are referred to as *targeted variables*.

**Table 2:** Summary of the predicted targeted variables.

Targeted variables	Values	Analysis Type
Extrusion temperature	HME temperature (°C)	Regression
Filament mechanical characteristics	Unextrudable, Flexible, Good or Brittle	Multi-classification
Printing temperature	Printing temperature (°C)	Regression
Printability	Yes or No	Binary Classification

Regression analyses were performed to predict HME temperature and FDM printing temperature, since the targeted variables were continuous numerical values. Classification analyses were performed to predict the filament mechanical characteristics and printability (Elbadawi et al., 2020). The labels used for filament mechanical behavior were either ‘good’, ‘brittle’, ‘flexible’ or ‘unextrudable’ based on the comments found in the reported studies. Good filament referred to a filament with mechanical behavior similar to commercial filaments. A brittle filament was defined as one that was susceptible to fracturing when it was bent from 180° to 90°. A flexible filament was one that easily bent when held from one side, due to a lack of structural integrity. Filaments that could not be obtained by HME, even when tested over a wide range of HME temperatures, were labelled as unextrudable. Printability was qualitatively classified as either ‘Yes’ or ‘No’ depending on whether the filament was able to be extruded through the nozzle of the FDM printer given the selected printing parameters.

### 2.5 Machine learning techniques

A computer running an Ubunto 20.04.2 LTS operating system, with an Intel® Xeon® CPU E5620 (2.40 GHz) CPU and an installed RAM memory of 32 GB, was used for data analysis and development of ML models as described below.

Three machine learning techniques (MLTs) were used: *artificial neural networks* (ANN) (Nagy et al., 2019), *support vector machines* (SVM) (Noble, 2006; Wang et al., 2022), and *random forests* (RF) (Belgiu and Drăguţ, 2016). Explanation on each MLT can be found in our previous study (Elbadawi et al., 2020). These were developed using *python* (version 3.8.10) with the Scikit-Learn package (scikit-learn, v1.0.1). A 75:25 split was used for training and testing the MLTs.

Before training any machine learning model, any formulation with missing data was removed. Both numerical and categorical input data was pre-processed and transformed (Nawi et al., 2013). Quantile transformation was applied to numerical variables for them to have a Gaussian distribution, which is known to have a positive impact in the performance of the trained machine learning models. On the other hand, categorical variables were label encoded, which simply replaces each possible categorywith a unique number.

In addition to the feature sets described in Section 2.2, other process-related parameters such as the extrusion or printing speed were also used to develop the ML models. Each possible set of process-related parameters was evaluated on the MLTs using all the feature sets described in Section 2.2 using a 50-random cross validation process. This was done for each target variable, as described in Section 2.4. The set of process-related parameters, feature set and MLT that gave the best performance (as described in Section 2.6) was obtained.

Upon identifying the best performing combination of process-related parameters, feature sets, and MLT, the best set of hyper-parameters for each algorithm was determined. A fixed set of possible values for each hyper-parameter was pre-defined (Table S1). Then, each possible combination of values for the hyper-parameters was tested using a 10-fold cross validation process.

### 2.6 Data evaluation

The performance of each MLT was evaluated based on numerous metrics depending on the type of analyses being conducted. A brief explanation of each metric can be found in our previous study (Elbadawi et al., 2020). For classification analyses, five classification metrics were used: *accuracy*, Cohen's *kappa*, *precision*, *recall*, and *F1*. For the processing temperature and dissolution time predictions, two regression metrics were used: the *mean absolute error* (MAE) and the *coefficient of determination* (R^2^).

## 3 Results & discussion

### 3.1 Exploratory data analysis

**Fig. 3:**
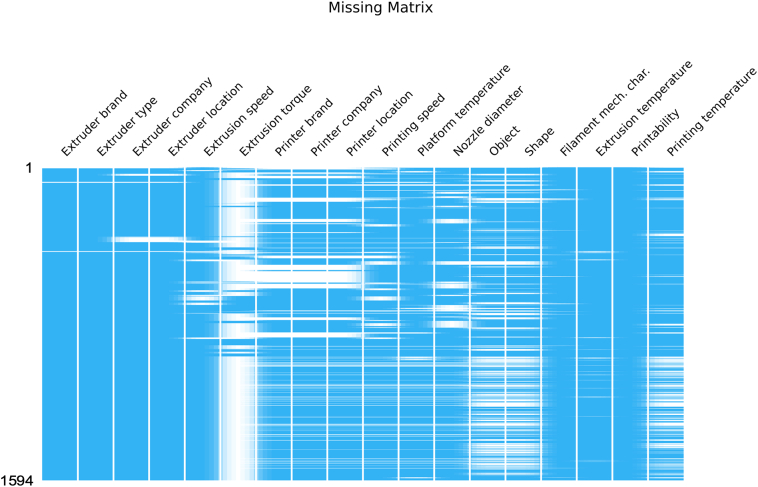
Diagram representing the dataset, used to illustrate the missingness of the data for each of the 1594 formulations. Blue indicates information was available, whereas white areas indicate missing data. (For interpretation of the references to color in this figure legend, the reader is referred to the web version of this article.)

An exploratory data analysis was performed to detect anomalies and identify the data pre-processing steps necessary for better machine learning performance. The combined dataset, comprising in-house and literature mined formulations, consists of 1594 formulations and 260 materials. This combination produced a more diverse dataset, with a broader variety of formulations and range of printing outcomes. Conversely, the diversity of materials remained relatively unchanged, since most materials used in in-house formulations were also used in formulations found in published literature. As shown in Fig. 3, the availability of these process-related parameters in the dataset is very heterogeneous. As formulations with missing data must be removed yet information about each formulation needs to be retained as much as possible, a balance between the number of parameters included as input and the amount of data preserved for training was evaluated. To do so, an exhaustive analysis was performed to determine the optimal set of process-related features. To minimize the loss of data, any combination that resulted in a loss greater than 25% of the original number of formulations was disregarded.

**Fig. 4:**
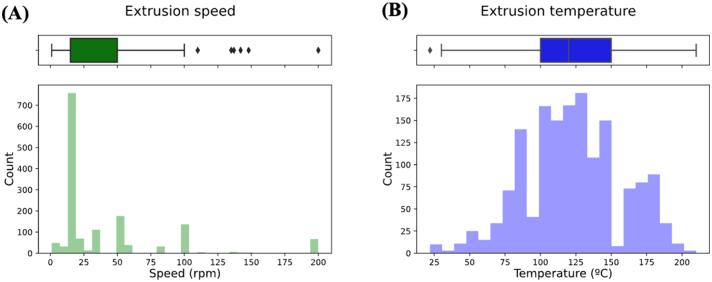
Histograms depicting distribution of (A) extrusion speed and (B) extrusion temperature.

Analysis of HME parameters revealed that 93.1% of formulations had the extrusion speed used for preparing filaments reported, of which 49.4% of filaments were extruded at a speed of 15 RPM. Extrusion speed ranged between 1 and 200 rpm (Fig. 4A). In contrast, only 15.0% of data reported extrusion torque, and was therefore excluded from analysis. The lack of reporting on extrusion torque values is likely due to the inability to conveniently measure the parameter on the most used hot melt extruders. The extrusion temperature used for HME ranged from 22 °C to 210 °C, with a mean of 123.8 °C (Fig. 4B).

**Fig. 5:**
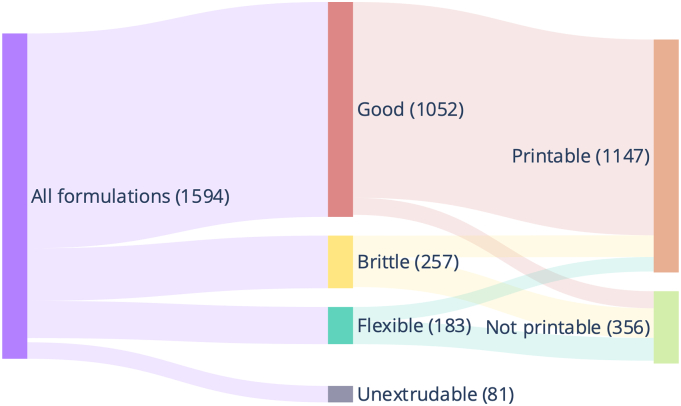
Sankey diagram showing distribution of extrusion and printing outcomes.

66.0% of formulations resulted in filaments with mechanical characteristics that were described as “Good” (Fig. 5). This represents a more balanced dataset compared to that derived from literature-mined formulations only, with 84.6% of the latter reporting “Good” filaments. As observed in the previous study, most filaments with “Good” mechanical characteristics were printable, while printing outcomes with “Brittle” and “Flexible” filaments were almost evenly split (Fig. 5).

**Fig. 6:**
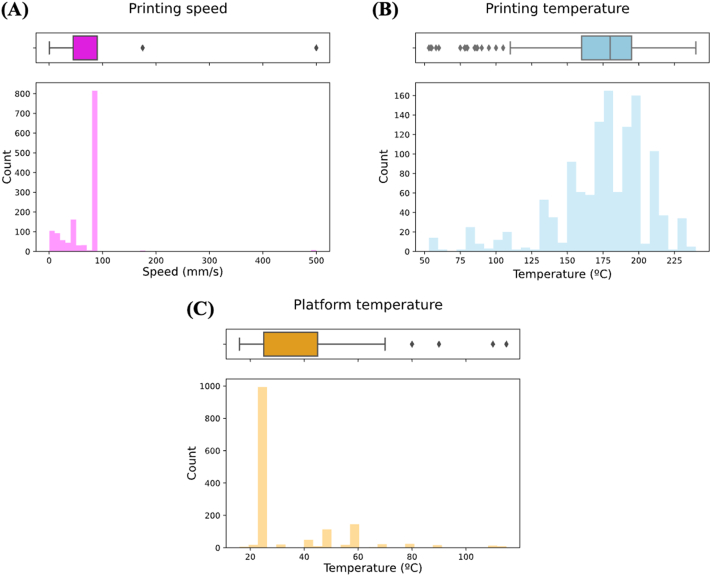
Histograms depicting distribution of (A) printing temperature, (B) platform temperature, and (C) printing speed.

Amongst formulations that were extrudable (i.e., the filaments were either “Good”, “Brittle” or “Flexible”), 81.2% of the derived filaments were fed through FDM 3D printers equipped with 0.4 mm diameter nozzles. The size of nozzle diameter ranged from 0.2 to 0.5 mm. The printing speed ranged between 0.5 mm/s to 500 mm/s, with 90 mm/s being the most used printing speed (52.9% of filaments fed into an FDM 3D printer). As illustrated by the boxplot, printing speeds above 100 mm/s represent a small minority of tested formulations (Fig. 6A). These were nonetheless included in analysis as they were actual trials and do not represent statistical outliers. The FDM printing temperature used ranged from 53 °C to 240 °C, with a mean of 174.3 °C. Likewise, the boxplot depicts a notable number of outliers at temperatures below approximately 110 °C (Fig. 6B). As this information may benefit researchers investigating printing at low processing temperatures for thermally labile drugs, these were retained in the dataset. The platform temperature used, which affects printing outcomes by influencing the adhesion of feedstock onto the platform, ranged between 16 °C to 115 °C, with a temperature of 25 °C (room temperature) being most used (Fig. 6C). 71.6% of formulations reported in the dataset used in this study were printable, compared to 85.7% in the literature-mined dataset. The greater diversity in HME and printing outcomes compared to previously used datasets provides more negative samples for MLTs to learn from, conceivably leading to better prediction performance.

### 3.2 MLT performance in predicting target variables

#### 3.2.1 Filament mechanical characteristics results

**Fig. 7:**
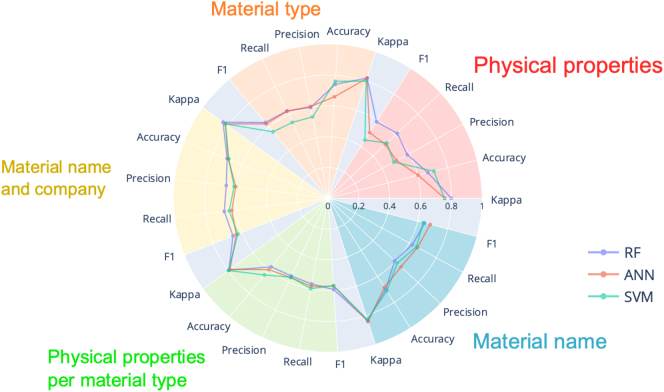
Radar plot with metrics results for filament mechanical characteristics. RF – random forests, ANN – artificial neural networks, SVM – support vector machines.

For predicting the mechanical characteristics of extruded filaments, the algorithm that performed the best was RF (Fig. 7). The best feature set was *material name* and the optimal set of process-related parameters included were *extruder brand*, *extruder type*, *extruder company*, and *extrusion temperature.*

The optimal hyper-parameters obtained for the random forest are shown in Table 3. The optimized RF model obtained the following scores in the final evaluation: an *accuracy* of 84%, a *Cohen's kappa* of 0.69, an *f1 score* of 0.72, a *recall* of 0.69 and a *precision* of 0.75. The RF model obtained in this study outperformed the optimized model using only in-house data, in which the highest obtained accuracy and *kappa* value were 73% and 0.61, respectively. Additionally, the current model attained a higher *kappa* value than the model optimized using literature-mined data alone (*κ* = 0.49), although the accuracy obtained was lower than that of the latter (accuracy = 91%). However, considering that the literature-mined data represents a more imbalanced dataset, comparing the accuracy of the two models does not correctly reflect their relative performance. With the literature-mined data containing a larger percentage of filaments with “Good” mechanical characteristics (as described in Section 3.1), a model built on this dataset that simply assigns all formulations as “Good” would naturally obtain a higher accuracy score than a model built on the combined dataset. Therefore, using the *kappa* value, which factors in the probability of chance agreement (i.e., a baseline value), would provide a better comparison of the performance of the two optimized models. As such, the results demonstrate that the use of a larger and more diverse dataset results in improve performance by machine learning models.

**Table 3:** Optimal hyper-parameters for random forest predicting the respective target variables.

Parameter	Filament mechanical characteristics	Extrusion temperature	Printing temperature	Printability
Bootstrap	False	False	False	False
Maximum depth for trees	Unlimited	Unlimited	Unlimited	Unlimited
Maximum features for trees	√n	√n	√n	√n
Minimum samples for leafs	1	1	1	1
Minimum samples for split	5	2	2	5
Number of trees	400	800	1000	400

#### 3.2.2 Extrusion temperature results

**Fig. 8:**
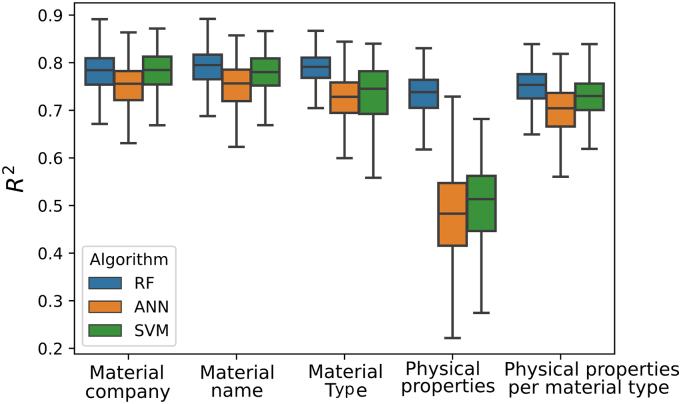
R^2^ for extrusion temperatures using the different ML techniques and feature sets (horizontal axis). RF – random forests, SVM – support vector machines, ANN – artificial neural networks.

When predicting extrusion temperature, only formulations derived from filaments with mechanical characteristics that were recorded as “Good” were used. RF was the best performing MLT for predicting the extrusion temperature, obtaining the highest *R*^*2*^ amongst the MLTs explored using all five feature datasets (Fig. 8). The best prediction results using RF were obtained when using the *material name* feature set together with the following set of process-related parameters: e*xtruder brand*, e*xtruder type*, e*xtruder company and extruder location*.

The optimal values for each hyperparameter for the random forest algorithm to predict the extrusion temperature are shown in Table 3. Subsequently, the optimized RF model obtained a MAE of 5.54 °C and an R^2^ of 0.91. This model outperformed the previous model built on in-house data alone, which obtained a MAE and R^2^ of 10.8 °C and 0.56, respectively. However, while the current model achieved a higher R^2^ than the model built on only literature-mined data (R^2^ = 0.90), the lowest MAE obtained by the current model is larger than that of the latter (MAE = 5.18 °C). This suggests that, although the difference between the two is small, the new model is less accurate in the small-scale (less than 1 °C) but makes fewer large-scale errors, which are more penalized by R^2^.

#### 3.2.3 Printing temperature

**Fig. 9:**
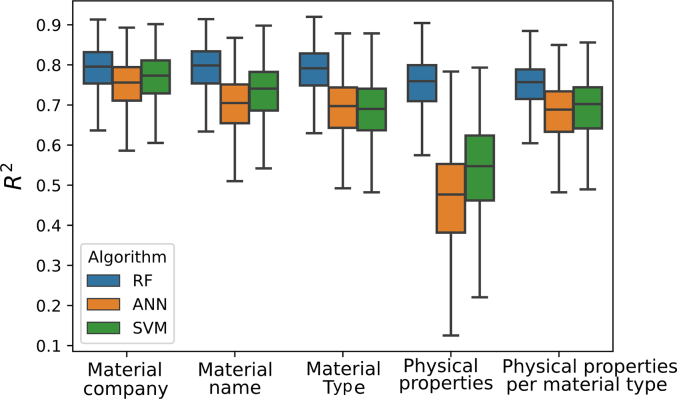
R^2^ for printing temperatures using the different ML techniques and feature sets (horizontal axis). RF – random forests, SVM – support vector machines, ANN – artificial neural networks.

Akin to extrusion temperature predictions, only formulations that were printable (i.e., labelled as “yes” for printability) were used for building the prediction models for printing temperatures. RF performed the best in predicting printing temperature (Fig. 9), outperforming the other models tested using all five feature sets. The feature set that provided the best prediction was *material with company name* and the selected process-related parameters were *printer brand*, *printer company*, *printer location*, *object,* and *shape.*

The hyperparameter values that provided the best performance using RF are shown in Table 3. The optimized random forest model obtained a MAE of 5.99 °C and a R^2^ of 0.88 in the final evaluation. The current model outperformed the performance of models built on in-house data alone (R^2^ = 0.83, MAE = 8.4 °C) and those built on literature-mined data only (R^2^ = 0.86, MAE = 6.87 °C). Considering that the printing temperatures ranged between 53 and 240 °C, the ability to predict the optimal printing temperature within ±5.99 °C is impressive and would yield significant time saving in formulation and printing development.

#### 3.2.4 Printability

**Fig. 10:**
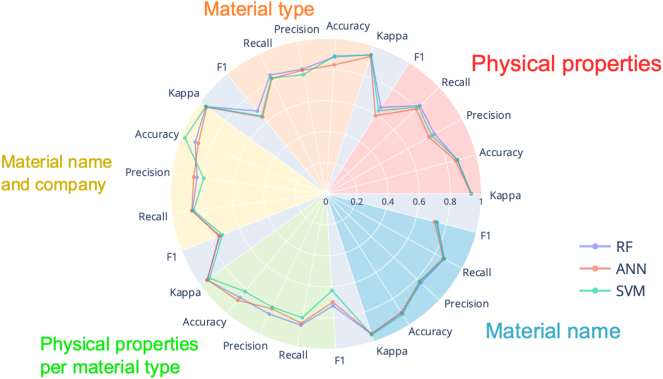
Radar plot with metrics results for printability. RF – random forests, ANN – artificial neural networks, SVM – support vector machines.

Once again, RF provided the best performance for predicting the printability of formulations (Fig. 10). The feature set that produced the best performance metrics was *physical properties per material type* together with the following set of process-related parameters: *printer brand, printer company, printer location, printing speed, object, and shape*.

The results of the hyper-parameter optimization process for the algorithm are shown in Table 3. The trained random forest obtained the following scores in the final evaluation: an *accuracy* of 84%, a *Cohen's kappa* of 0.66, a *f1 score* of 0.80, a *recall* of 80% and a *precision* of 81%. This outperformed the model that used in-house data only (*κ* = 0.52) and that using literature-mined data only (*κ* = 0.56). This further supports the need for balanced datasets for machine learning models to provide accurate and reliable predictions.

### 3.3 General considerations

**Fig. 11:**
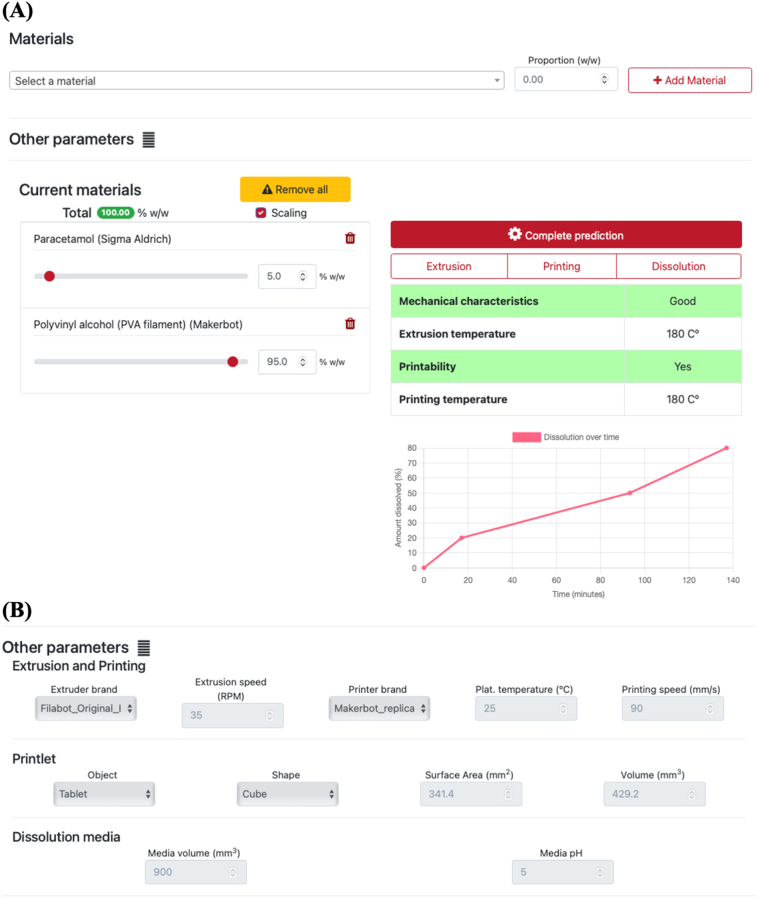
Screenshot of (A) landing page of web application, and (B) input parameters that users may manipulate upon clicking the drop-down menu.

The optimized ML models were integrated in a web application service that is easily accessible from any device with internet connectivity via the following link (https://m3diseen.com/predictionsFDM/) (Fig. 11). The application is hosted on a standard server, using the open-source software Caddy 2 for serving a web application written in Python3 using the Django web framework. The web application and web server modules run as Docker containers on a Docker server (version 20.10.7) The machine learning models were integrated and functionalized using the scikit-learn package.

On the web application, users will be prompted to select the material they are using from a pre-defined list and specify the proportion (in % *w*/w) included in the formulation. Users may add materials until the formulation composition reaches 100% w/w, for which the color of the bubble around the total weight composition (under the “Current materials” tab) will turn green. Entering a formulation composition that is more or less than 100.00% w/w will disable the “Complete prediction” button. Users may also access and specify other parameters that may influence prediction by clicking the drop-menu adjacent to the “Other parameters” heading. These parameters include the extruder brand, the object and shape to be printed, and the dissolution media volume and pH. Upon clicking “Complete prediction”, the filament's mechanical characteristics, extrusion temperature, printability, printing temperature, and dissolution profile of the resulting object, will be provided in the bottom right window. Dissolution predictions are based on the literature-mined only dataset, as reported in our previous work (Muñiz Castro et al., 2021). The web application was able to provide all 5 predictions within 5.76 ± 1.24 ms. As such, this easily accessible platform provides quick remote feedback on the performance of experimental formulations, affording researchers considerable time savings in formulation development and optimization.

This study integrated data from published articles and in-house data to produce a more balanced dataset, which was subsequently used to produce ML models that performed better than those created in previous studies. Due to the input of more negative data (especially with regards to filament mechanical characteristics), higher predictive performances were obtained in this study. Further improvement in performance is therefore expected with more negative data, especially on filaments that were not printable. As data is obtained from a range of laboratories using different equipment and methodologies, the primary challenge would be to standardize reporting so that the degree of missingness in the overall dataset may be reduced. At the minimum, we implore authors to report the parameters enumerated in Table 1, although additional information such as the size of the printed object and the dissolution parameters may be useful for future applications. Future work will aim to build a platform wherein pharmaceutical 3DP researchers may easily share their data in a comprehensive and structured manner.

Random forest emerged as the best MLT for predicting all targeted variables in this study. However, this finding may, and will likely, not be the case for every dataset containing FDM 3DP and HME formulations. Every MLT has their own advantages, and the best performing MLT might change as the dataset mutates. For regression analyses (extrusion temperature and printing temperature), there is a reason why RF performed particularly well. As a tree-based algorithm, RF lacks extrapolation. Predicted values given by tree models are the mean of a set of previously seen training examples that share some pattern with the input, which makes them unable to predict values outside the distribution in which they have been trained. This, which is normally a disadvantage, may have turned out to be an advantage in the case of our dataset. Extrusion and printing temperatures, vary in a relatively small range, and tend to repeat round values like 115 °C, 120 °C, 125 °C, etc. For example, we have more than 100 formulations with an extrusion temperature of 100 °C and more than 60 with 105 °C, but no formulation between 101 °C and 104 °C. When evaluating the predictions against a test set, RF output is often very close to previously seen round values, in this case usually matching test set values, while ANN output approximates a more continuous distribution. As the dataset grows, we expect ANN to become a feasible technique to be employed to give the best predictive performance, given that it has consistently outperformed MLTs such as RF and SVM (Castiglioni et al., 2021; Zhang et al., 2017). However, a large dataset on the scale of tens of thousands to millions of data points is necessary, which in turn necessitates further formulation attempts and open reporting within the pharmaceutical 3DP community. While it is understandable that the value of reporting technical failures might not be immediately apparent, the ever-increasing calls for open and transparent scientific reporting could make publishing negative data common practice. As a step in this direction, we encourage fellow pharmaceutical 3DP researchers to publish negative data as supplementary materials or forward their negative data to us (a.goyanes@fabrx.co.uk), so that the models integrated in the web application may be gradually improved.

To foster open research within the pharmaceutical 3DP research community, we have integrated the optimized ML models from this study into an open-source web application that provides prediction on filament mechanical characteristics, extrusion and printing temperatures, printability, and drug release profiles. Due to the absence of dissolution data from in-house formulations, prediction of drug release profiles is based on models built from our previous work (Muñiz Castro et al., 2021). Free online tools for accelerating formulation developments are also being developed elsewhere by large pharmaceutical and excipients companies. For example, BASF's ZoomLab™ uses a proprietary algorithm to provide predictions on the most effective formulations based on the user's chosen active ingredient and desired product profile (BASF, 2022). *M3DISEEN* represents the first and, at the date of writing, only web-based service for predicting FDM 3DP formulation performance. These existing online tools demonstrate the critical role that Artificial Intelligence will play in accelerating and supporting research in the pharmaceutical industry. As 3DP is gradually adopted as an alternative to conventional pharmaceutical manufacturing, it is hoped that ML will be capable of simulating the entire 3DP workflow and provide “backward” predictions akin to BASF's ZoomLab (i.e., providing formulation suggestions based on the desired physicochemical and dissolution properties).

## 4 Conclusion

In this study, in-house and literature-mined data on HME and FDM 3DP formulations were successfully used to provide enhanced ML predictive performance compared to those achieve in previous works. The dataset comprised 1594 formulations with more heterogenous hot melt extrusion outcomes. The optimized ML models were able to predict printability and filament characteristics with higher accuracies, and HME and FDM printing temperatures within narrower temperature ranges than previous iterations. *M3DISEEN*, the web-based tool for guiding HME and FDM 3DP formulation development, was updated with the new ML models for predicting filament mechanical characteristics, printability, extrusion & printing temperatures, and drug release profiles. This study demonstrates the importance of having a balanced dataset for optimal ML performance. In this vein, accelerating research in pharmaceutical FDM 3DP through ML is arguably hampered by the lack of negative data. Therefore, we encourage pharmaceutical 3DP researchers to publish data on failed prints or forward their negative data to us so that *M3DISEEN* may be gradually improved. With open and standardized reporting of data, new and reliable knowledge may be generated using ML to advance pharmaceutical 3DP into clinics.

## CRediT authorship contribution statement

**Jun Jie Ong:** Conceptualization, Data curation, Formal analysis, Investigation, Methodology, Writing – original draft, Writing – review & editing. **Brais Muñiz Castro:** Conceptualization, Data curation, Formal analysis, Methodology, Visualization, Writing – original draft. **Simon Gaisford:** Supervision. **Pedro Cabalar:** Supervision. **Abdul W. Basit:** Supervision, Resources, Writing – review & editing. **Gilberto Pérez:** Supervision, Writing – review & editing. **Alvaro Goyanes:** Conceptualization, Methodology, Project administration, Resources, Supervision, Writing – review & editing.

## Declaration of Competing Interest

The authors declare that they have no known competing financial interests or personal relationships that could have appeared to influence the work reported in this paper.

## Appendix A Supplementary data

Supplementary material: Parameter grid for machine learning techniques Image 1
