# Supplementary material for: Accelerating 3D printing of pharmaceutical products using machine learning
Source: Int J Pharm X. 2022 Jun 9;4:100120. doi: 10.1016/j.ijpx.2022.100120 (PMC9218223; doi:10.1016/j.ijpx.2022.100120)
Supplement: Supplementary file 1 — Supplementary material: Parameter grid for machine learning techniques [file mmc1.docx]

**Supplementary materials for “*Accelerating 3D Printing of Pharmaceutical Products using Machine Learning*”**

Jun Jie Ong^1,a^, Brais Muñiz Castro^2,a^, Simon Gaisford^1,4^, Pedro Cabalar^2^, Abdul W Basit^1,4*^, Gilberto Pérez^2,*^, Alvaro Goyanes^1,4,5*^

^1^ Department of Pharmaceutics, UCL School of Pharmacy, University College London, 29-39 Brunswick Square, London WC1N 1AX, UK.

^2^ IRLab, CITIC Research Center, Department of Computer Science, University of A Coruña, Spain

^3^ IRLab, Department of Computer Science, University of A Coruña, Spain.

^4^ FabRx Ltd., 3 Romney Road, Ashford, Kent, TN24 0RW, UK.

^5^ Departamento de Farmacología, Farmacia y Tecnología Farmacéutica, I+D Farma (GI-1645), Facultad de Farmacia, iMATUS and Health Research Institute of Santiago de Compostela (IDIS), Universidade de Santiago de Compostela, 15782, Spain.

^a^ These authors contributed equally to this work.

* Correspondence: a.basit@ucl.ac.uk (A.W.B.); a.goyanes@fabrx.co.uk (A.G.), gilberto.pvega@udc.es (G. P),

| Table S1. Parameter grid for MLTs | |
| --- | --- |
| **Parameter** | **Possible values** |
| ***Random forest*** | |
| Bootstrap | False, True |
| Maximum depth for trees | 7, 15, Unlimited |
| Maximum features for trees | √n |
| Minimum samples for leafs | 1, 2, 4 |
| Minimum samples for split | 2, 5, 10 |
| Number of trees | 200, 400, 600, 1000, 1600 |
| ***Support vector machine*** | |
| C | 10^-2^, 10^-1^, 1, 10 |
| Gamma | 10, 1, 10^-1^, 10^-2^, 10^-3^, 10^-4^ |
| Kernel function | RBF, polynomial, sigmoid |
| ***Artificial neural network*** |  |
| Hidden layer sizes | (50,10), (60,10), (100, 50, 10) |
| Activation function | TANH, RELU |
| Solver | SGD, adam |
| Alpha | 10^-4^, 10^-3^,10^-2^, 10^-1^, 1, 10 |
| Learning rate | Constant, adaptative |
